# Surface Texture of Macroplastic Pollution in Streams Alters the Physical Structure and Diversity of Biofilm Communities

**DOI:** 10.1111/1758-2229.70068

**Published:** 2025-04-10

**Authors:** Fabiola Lopez Avila, Krista A. Capps, Raven L. Bier

**Affiliations:** ^1^ Odum School of Ecology University of Georgia Athens Georgia USA; ^2^ Savannah River Ecology Laboratory University of Georgia Aiken South Carolina USA

**Keywords:** freshwater biofilms, macroplastic, microbial biofilms, plastic pollution

## Abstract

Biofilms can develop on nearly any surface, and in aquatic ecosystems they are essential components of biogeochemical cycles and food webs. Plastic waste in waterways is a new type of surface for biofilm colonisation. To analyse the influence of plastic pollution on the development and diversity of microbial freshwater biofilms that colonised them, we incubated 388 cm^2^ veneers of high‐density polyethylene (HDPE) with two veneer textures, smooth and rough, and tulip tree wood ( *Liriodendron tulipifera* ), in three rural headwater streams at the Savannah River Site (Aiken, SC, USA). We collected biofilms from veneers after 14, 28 and 56 days of incubation and analysed 16S rRNA genes and biofilm properties. We found that plastic negatively affected species richness of biofilms compared with wood, but that evenness was greatest on rough textured HDPE. Beta diversity was primarily influenced by stream site. Beta diversity differed more between wood and plastic veneers than with plastic surface texture and became more different over time. Wood had nine times more biomass than rough HDPE and 40 times more biomass than smooth HDPE. Given the projected increase of macroplastic pollution in aquatic ecosystems, our findings emphasise the need to further understand its effects on biofilm characteristics.

## Graphical abstract

Biofilms develop on natural surfaces in stream ecosystems, but increasingly unnatural surfaces such as plastic trash are also available. In a field experiment, biofilms on wood veneers or plastic veneers with a smooth or rough surface texture differed in bacterial alpha and beta diversity and biofilm biomass. These differences may have consequences for the functional contributions of biofilms to stream ecosystems.

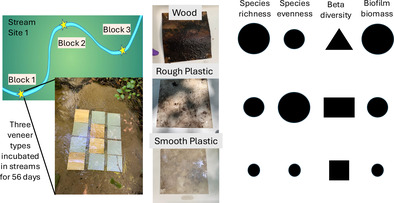

## 1 Introduction

Biofilms are an essential part of aquatic ecosystem structure and function, providing habitat, supporting food webs, and facilitating biogeochemical cycling (Battin et al. 2016; Vosshage, Neu, and Gabel 2018). Biofilm structure, composition, and function are substrate‐specific and change with time (Wilhelm et al. 2014; Hellal et al. 2016). The development of biofilms can also be influenced by the physical surface, or surface texture, of the substrate as rough surfaces are known to provide anchoring points for biofilm cell attachment (Romaní 2010). Anthropogenic activities alter the physical distribution and abundance of hard substrates in streams. For example, the loss of large woody debris and other riparian vegetation from watersheds (Fuller et al. 2022) reduces the amount of wood substrates moving into streams, and the addition of discarded items, such as plastic trash, introduces novel substrates into freshwater environments (Hoellein and Rochman 2021). This change in substrate availability both creates novel surfaces for biofilm development and reduces the total amount of natural substrate habitat.

Since 1950, plastic production has exponentially increased, and is predicted to continue increasing (Rouch 2023). Nearly 80% of plastic waste accumulates in landfills or the natural environment with the total amount of unreclaimed plastic expected to amount to 12,000 million tons by 2050 (Geyer, Jambeck, and Law 2017). Large proportions of this plastic end up in rivers where the majority is stored after deposition and subsequent sedimentation, or it is conveyed to inland lakes or oceans (van Emmerik et al. 2022). Estimates suggest that between 1 and 2.4 million tons of plastic enter the ocean each year through river networks (Lebreton et al. 2017). Plastic pollution can be found in many areas of a stream: throughout the water column, sediment, pools, and riffles (Lenaker et al. 2019; Cowger et al. 2021; Haberstroh et al. 2021) and has the potential to serve as a substrate for biofilm development in each of these habitats. Many freshwater studies of plastic influence have occurred in urban streams (e.g., Lazcano, Kelly, and Hoellein 2024; Vincent et al. 2022), yet as plastic production and litter expands with development, previously unimpacted areas are likely to see the introduction of macroplastic pollution into stream ecosystems.

There are many types and morphologies of plastic trash that may influence the structure and diversity of microbial communities that colonise plastic surfaces. For instance, Wang et al. (2021) found that chemically distinct compositions of plastic (e.g., polyethylene compared with polypropylene) influence the richness of bacterial communities. Similarly, Pinto et al. (2019) found that the type of plastic polymer influenced biofilm colonisation and bacterial community composition on plastic in the ocean, especially in the initial stages of colonisation.

There is some evidence that the surface texture of plastics may also influence microbial community composition in biofilms. For example, in a freshwater, urban river, Lazcano, Kelly, and Hoellein (2024) found that more complex and rougher surfaces from different plastic types can positively influence diversity in microbial communities relative to communities found on smooth surfaces. They found that species richness and Shannon diversity of bacteria and algae were highest in biofilms on wood and polystyrene foam when compared to two forms of low‐density polyethylene: one flexible, translucent, plastic film and the other a rigid, opaque, sheet. Studies have also used ultraviolet light to weather plastics which, in one study, increased surface roughness and led to higher richness and lower evenness of prokaryotic biofilms after 72 h compared with untreated plastics (Rummel et al. 2021). However, they also found that ultraviolet light treatments increased surface hydrophobicity and altered the stream water dissolved organic matter compound classes they adsorb, suggesting that the influence was not due solely to roughness. Several studies have shown that surface texture may influence bacterial adhesion to a given surface (Dika et al. 2013; Ammar et al. 2015; Bohinc et al. 2016). Renner and Weibel (2011) suggested that surface roughness enhances the adhesion of bacteria to substrates because it provides more surface area for the cell to attach, and it reduces the shear force experienced by microorganisms in flowing habitats.

Interactions between the chemical composition of a substrate surface and surface texture have also been examined in a limited number of studies. For instance, Lorite et al. (2011) found hydrophobic and rough surfaces did not influence the development of a single‐species biofilm as strongly as the chemical composition of the plastics. They studied the colonisation of *X. fastidiosa* , a gram‐negative bacterium, on glass and silicone substrates. They found that *X. fastidiosa* cell adhesion was a result of the conditioning film, or macromolecules on the substrate, rather than surface roughness or hydrophobic surface properties. This study shows an example of a single‐species biofilm; however, much remains to be learned about the consequences of interactions between substrate type and substrate surface for the composition of multi‐species microbial communities in biofilms of natural environments.

In this study, we asked how macroplastic (plastic > 2.5 cm) pollution influences the development and composition of biofilm microbial communities in unpolluted, rural, freshwater stream ecosystems relative to a natural wood substrate and how the surface texture of chemically identical plastic polymers modify this development. We used 16S rRNA genes to examine how species diversity and taxonomic identity of microbial biofilms colonising different veneer types—plastic veneers and wood veneers—differed from one another over 56 days of incubation in headwater streams. We also investigated how the surface texture of plastic influenced the diversity of biofilm communities colonising hard substrates in streams. Finally, we sought to understand how substrate type and surface texture influenced the mass and chlorophyll‐*a* content of these biofilms. We hypothesized that if microbial communities from rural stream ecosystems are maladapted to plastic substrates, then the number of species able to colonise plastics would be lower than on a non‐plastic substrate, and this would result in a change in species diversity and species evenness in the biofilms. We also expected that the veneer texture would influence species diversity and evenness in biofilms such that greater habitat complexity in rougher surfaces, including wood and distressed plastic, would create more diverse microbial communities than smooth, plastic surfaces.

## 2 Experimental Procedures

### 2.1 Site Descriptions and Sampling

**FIGURE 1:**
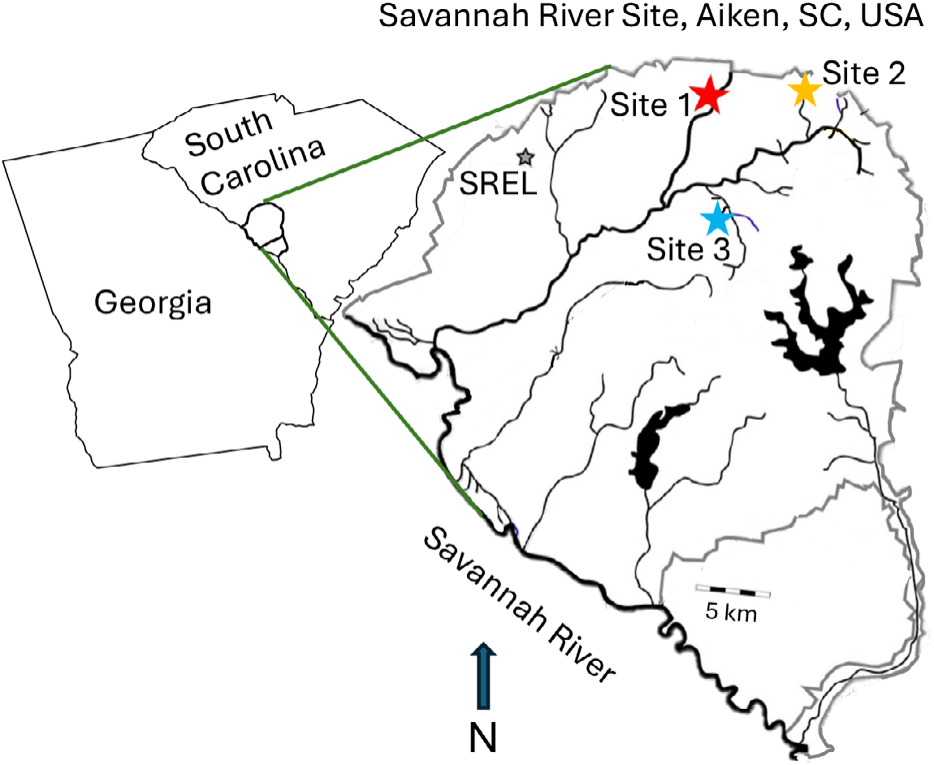
Study sites where the experiment was conducted in three headwater streams at the Savannah River Site, Aiken, South Carolina, USA.

We conducted a field experiment in three, rural headwater streams on Savannah River Site (SRS) (Aiken, SC, USA; Figure 1). The stream sites were first order (Strahler) streams at Upper Three Runs (Site 1), Tinker Creek (Site 2), and Mill Creek (Site 3) which are located in the Sand Hills ecoregion. They spanned 1–2 m in width and sampling locations were between 0.6 and 1.3 km downstream from the stream source. Streams were forested and shaded with intact riparian zones that are bottomland hardwood forests with cane (*Arundinaria* sp.), holly (*Ilex* sp.), sweet gum (*Liquidambar styraciflua*), and oaks (*Quercus* spp.) (Imm and McLeod 2005). No industrial facilities were upstream and no macroplastic pollution was detected likely due to the restricted access to Savannah River Site. On July 18, 2022, we deployed three experimental groups (blocks) in each stream, each containing 12 veneers with dimensions 19.7 cm × 19.7 cm × 1.5 mm (Figure S1). Each block contained four replicates of three veneer types: high‐density polyethylene (HDPE) with a smooth surface (smooth), HDPE with a distressed surface (rough), (HDPE sheets, United States Plastics Corporation, Lima, OH, USA) and untreated tulip tree wood ( *Liriodendron tulipifera* ) (Poplar wood sheets, Ocooch Hardwoods, Viola, WI, USA).

Our choices for the plastic polymer and wood were based on the prevelance of these surfaces. We selected polyethylene as the polymer type because it is one of the most commonly found polymers in the aquatic environments where this has been surveyed and is abundant in freshwater sediments and epipelagic zones (Schwarz et al. 2019; Hoseini and Bond 2002). The main application for HDPE is to create packaging products (Geyer, Jambeck, and Law 2017). Tulip tree wood was selected because it is a common and broadly distributed tree species east of the Mississippi River that, especially in the southern sites, grows along stream bottoms including at the Savannah River Site (Beck 1990; Imm and McLeod 2005).

We created the rough surface on veneers by using a handheld sander (Random Orbit Palm Sander, AR DEWALT brushless DCW2010) to scratch the surface of the smooth HDPE veneers with 60 grit sandpaper for 10 s with no additional pressure aside from the weight of the sander. Then, we secured each block to a metal grid using cable ties and anchored the grid to two submerged concrete masses such that veneers were submerged in ~2 cm deep of stream water. We spaced the blocks 10 m apart within the experimental reach. Each block included one additional replicate of each veneer treatment affixed to it to address the potential loss of veneers during deployment. We randomised the placement of veneers within each block into four groups, each having three veneer types (smooth, rough and wood). Then, we randomised the retrieval order of veneer groups, resulting in the collection of three smooth HDPE, three rough HDPE, and three wood veneers from each block in each stream during each sample day (Figure S1).

We recorded a subset of environmental characteristics in each experimental reach. Briefly, we measured dissolved oxygen, conductivity, water temperature and pH in situ using a hand‐held YSI ProDSS Multiparameter Digital Water Quality Meter (YSI Inc./Xylem Inc., Ohio). We recorded stream wetted width, depth and average water depth above veneer surfaces at deployment and collection dates. We also measured total organic carbon, chloride, nitrate, sulphate and phosphate from stream water grab samples that (except for total organic carbon samples) were filtered using a 0.2 μm filter. All samples were subsequently analysed at the University of Georgia Stable Isotope Ecology Laboratory.

We collected biofilm samples from submerged veneers on days 14, 28 and 56 after deployment using methods modified from Li et al. (2020). Following a storm event, a set of veneers was elevated above the water surface and the additional replicate was used instead. We removed the biofilm from each veneer by scraping the surface using a toothbrush and placed the samples in sterile Whirl‐pak bags on ice for transportation to the lab. In the lab, we filtered samples onto 0.2 μm pore size filters (Supor 200, PALL Corporation). Subsequently, we cut the filter in half and used one half to examine microbial community composition and the other half to estimate biofilm dry mass and chlorophyll‐*a* content.

### 2.2 Laboratory Processing

We stored half of the biofilm filter at −20°C until nucleic acid extraction with a DNeasy PowerWater kit (Qiagen, Germantown, MD) using the manufacturer protocol. We added a negative control to each batch of 24 extraction tubes. Next, we quantified the extracted DNA using a Quant‐iT PicoGreen dsDNA Assay Kit and a Synergy HTX Multi‐Mode Microplate Reader (BioTek). We sent the samples for 16S rRNA gene sequencing to the University of Delaware Sequencing & Genotyping Center where they amplified the DNA using primers 515F (Caporaso et al. 2011) and 805R (Herlemann et al. 2011) (V4 region). The PCR thermocycler conditions were 95°C for 3 min, with 25 cycles of 95°C for 30s, 60°C for 30s, 72°C for 30s, 72°C for 5 min. The centre sequenced amplicons with MiSeq PE 300 bp sequencing and included a negative and positive control (Illumina, San Diego, CA, USA).

The second half of filters were placed in foil‐covered 15 mL tubes and stored at −20°C until they were freeze‐dried to a constant mass for 1–2 days. This biofilm dry mass was weighed and multiplied by two to yield an estimate of biofilm dry mass for each veneer surface. After the mass was determined, we estimated chlorophyll‐*a* from the freeze‐dried biofilm using ethanol extractions as described by Kohler et al. (2022). We modified extraction methods slightly in that we placed the supernatant in microcentrifuge tubes before analysis to further separate particulates from the supernatant.

### 2.3 Bioinformatics

We generated demultiplexed sequence data for each of our samples. Each of the two sequencing plates had independent dereplication, filtering, trimming to 250 bp using cut adapt (version 17.1) (Martin 2011), error learning and sample inference. For sample processing, we used the R package ‘dada2’ to process paired‐end fastq files from the sequencer into merged, denoised, chimera‐free, inferred sample amplicon sequence variants (ASVs) (version 1.16) (Callahan et al. 2016) (Table S1). We assigned taxonomy of ASVs using the SILVA database with a 99% threshold (version 138.1) (Quast et al. 2013; McLaren and Callahan 2021), which had not yet implemented proposed changes in nomenclature. We processed the negative controls using the “decontam” package in R (Davis et al. 2018) and removed anything with a contamination score higher than 0.52, resulting in the removal of 114 contaminants. Additionally, we removed chloroplast, mitochondria and Eukarya ASVs from the dataset. We archived raw sequence data in the NCBI SRA (Sequence Read Archive) with accession BioProject number PRJNA1053619.

### 2.4 Statistical Analysis

We estimated the alpha diversity metrics richness, and Pielou's evenness using R software (version 4.1.2) (R Core Team 2022) using pooled stream sites. We assessed rarefaction curves (Figure S2) and then estimated richness using the package ‘breakaway’ (version 4.8.4) to determine the projected number of ASVs (Willis and Bunge 2015, 2017). Next, we estimated Shannon's index using package ‘metagMisc’ with function *phyloseq_mult_raref_div* and 999 iterations with a minimum number of 17,018 to match the lowest abundance of our samples. Then, we used estimated richness and Shannon index values to calculate Pielou's evenness. To estimate the effects of veneer characteristics and time on the heterogeneity of microbial community richness and evenness, we conducted mixed effects regression models using the R package ‘breakaway’ (version 4.8.4) with function *betta_random*. These functions use effects that can account for the heterogeneity of estimated diversity from multiple sites and calculate the significance of covariates and a hypothesis test. An additional random effect term in the models captures sample variability not explained by the covariates. We retained the richness and evenness models fit with the interaction between collection day and veneer type (smooth HPDE, rough HDPE and wood) as fixed effects and site as a random effect as those had better fits than models that excluded site (corrected Akaike information criteria: richness with site = 1247, without site = 1427; evenness with site = 355, without site = 521). Then, we tested the full model against a null model with a parametric bootstrap (9999 iterations) using function *test_submodel*, for an overall test of whether alpha diversity varied with surface type and time. In the event of significant overall test, we fit another model with *betta*_*random* such that a different veneer type served as the intercept so the diversity of each veneer type could be compared to the others. To determine the influence of collection day on the alpha diversity of each veneer type, we ran separate mixed effects models with collection day as the fixed effect and site as the random effect. S.E. represents the standard error from the mean. To evaluate the influence of site on alpha diversity, we conducted a three‐factor ANOVA with Bonferroni adjustment for richness using log‐transformed data and a Kruskal–Wallis test for evenness due to those data failing the Shapiro–Wilk test of normality even after transformations. For this, we used the ‘rstatix’ R package (version 0.7.2) with *anova_test* and *kruskal_test*, respectively (Kassambara 2023).

To understand the microbial groups associated with each veneer type and collection day, we conducted indicator taxa analysis using pooled stream sites and visualised patterns of community composition. For indicator taxa analyses, we used the phylogenetic order level and conducted the analysis with the *multipatt* function in R package ‘indicspecies’ with 999 permutations (version 1.7.14) (De Cáceres and Legendre 2009; De Cáceres et al. 2011). Our treatment groups for indicator taxa analysis were veneer type, collection day and the combination of these factors. We identified indicators as those with *p* < 0.05 based on the indicator value index, which is a product of taxon specificity and sensitivity to each treatment group. For community composition visualisations, we created bar plots of the major classes of bacteria and archaea using the top 500 most abundant classes.

We used ordination techniques to compare community composition across veneer type and sites and to identify environmental variables that were related to community structure. First, we removed ASVs occurring less than 10 times in the dataset and created a cumulative sum scaling normalised dataset with the ‘metagenomeSeq’ R package version 1.38.0 (Paulson, Pop, and Bravo 2013) to account for differences in library sizes. We used this dataset to visualise the beta diversity of microbial communities with a non‐metric multi‐dimensional scaling (NMDS) ordination of Bray–Curtis dissimilarities with 999 permutations using package ‘vegan’ (version 2.6‐4) (Oksanen et al. 2020). We fit environmental vectors with R function *envfit* in the ‘vegan’ R package. We conducted beta dispersion tests for collection day, site and veneer type to test for homogeneity of variability using the function *betadisper* in ‘vegan’. The collection day variable violated homogeneity of variances assumption (*p* = 0.015). We assumed that the lack of homogeneous variance in collection day could be caused by differences in location, dispersion or a combination of these. To evaluate differences in community composition among treatments and sites, we conducted a PerMANOVA test that included stream site and veneer type using the *adonis2* function in ‘vegan’ with 999 permutations (Anderson 2017). To aid in interpreting the PerMANOVA results, we calculated the differences of centroids within and between collection day group means in the NMDS to understand changes in variability among collection days.

To compare biofilm dry mass and chlorophyll‐*a* content among different veneer types, we pooled stream sites and conducted separate linear models for each response variable using the function *lm* in R package ‘stats’ (version 3.6.2) with veneer type and collection day as fixed effects. We then conducted post hoc Tukey contrast‐pairwise comparison tests using the *glht* function from the ‘multcomp’ package for veneer type (version 1.4‐25) (Hothorn, Bretz, and Westfall 2008). The *glht* function uses the single‐step method to adjust pairwise comparison *p* values.

## 3 Results

### 3.1 Alpha Diversity

**FIGURE 2:**
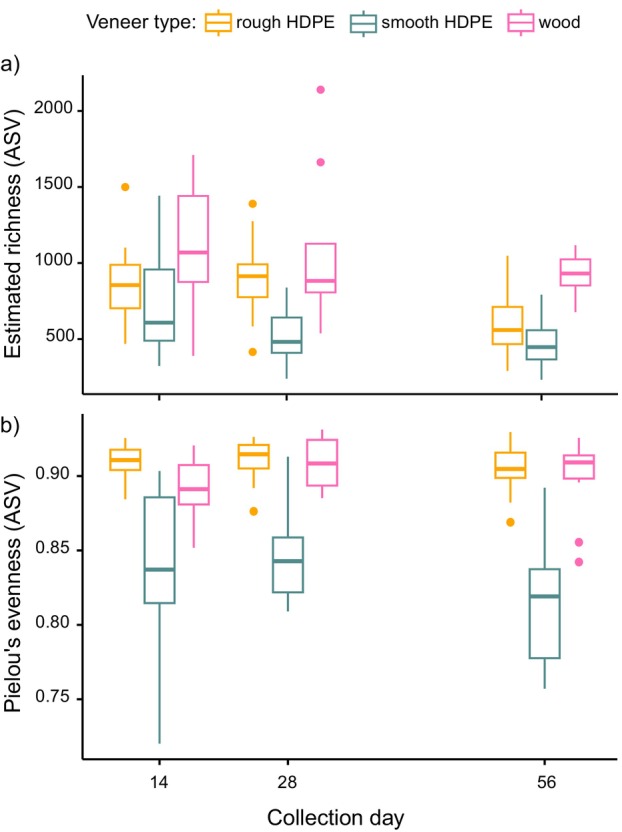
Alpha diversity metrics of freshwater microbial communities in biofilm collected from rough high‐density polyethylene (HDPE), smooth HDPE, and wood veneer types that were incubated in three sites for up to 56 days. (a) Estimated richness of microbial communities over time reported as amplicon sequence variants (ASVs). (b) Pielou's evenness of microbial communities over time (mixed effects regression models, *α* = 0.05, *N* = 3).

Microbial community alpha diversity metrics of richness and evenness were significantly influenced by the type and texture of veneers (wood, smooth HDPE and rough HDPE), but varied minimally with time (Figure 2). Wood veneers had the greatest estimated microbial species richness (1029 ASVs, S.E. = 74), followed by rough (801 ASVs, S.E. = 47), and then smooth (586 ASVs, S.E. = 46) plastic. Microbial community richness varied with veneer type and collection day (overall model F_4,95_ = 3.34, *R* ^2^ = 0.32, *p* < 0.001, Figure 2a, Table S2, Figure S3). Compared to wood veneers, on average, the model showed that smooth veneers had 382 fewer ASVs (S.E. = 83, *p* < 0.001). Although rough veneers had on average 139 fewer unique ASVs than wood veneers, this difference was not significant (S.E. = 83, *p* = 0.09). The texture of the plastic veneers influenced microbial community richness: rough HDPE had on average 243 more unique ASVs than did smooth HDPE (S.E. = 83, *p* = 0.003). Richness also differed by collection day, but the influence of time on richness was negligible and decreased richness by fewer than four ASVs (S.E. = 1, *p* = 0.004). Microbial community richness declined over time on each veneer type, but very few unique ASVs were lost. Rough veneers lost an average of 6 ASVs (S.E. = 2, *p* < 0.001). Both smooth and wood veneer communities lost an average of five ASVs (smooth S.E. = 2, *p* = 0.001, wood S.E. = 2, *p* = 0.013).

Community evenness also varied with the veneer type and time (overall model F_4,95_ = 37.8, *R* ^2^ = 0.53, *p* < 0.001, Figure 2b, Table S2, Figure S3). Community evenness was greatest on rough HDPE (0.91, S.E. = 0.003), followed by wood (0.90, S.E. = 0.004) and smooth HDPE (0.83, S.E. = 0008). Evenness declined in all treatments throughout the experiment. Greatest declines occurred on smooth veneers (0.12%, S.E. = 0.02, *p* < 0.001), whereas declines were minimal (0.03%) on rough and wood veneers (rough S.E. = 0.006, *p* < 0.001, wood S.E. = 0.009, *p* < 0.001).

In addition to veneer type and time, site was also an influential factor for community richness (three‐factor ANOVA, Site: F_2,81_ = 12, *p* < 0.001; Veneer type: F_2,81_ = 25, *p* < 0.001; Collection day: F_2,81_ = 12, *p* < 0.001;), but not for community evenness (Kruskal–Wallis, *H* = 4.67, *p* = 0.97). Stream Site 1 had significantly greater richness than the other two sites with an average richness of 975 ASVs, (S.E. = 66) while Site 2 had an average of 697 ASVs (S.E. = 60) and Site 3 had an average of 712 ASVs (S.E. = 51) (pairwise comparison Site 1 vs. Site 2: adj. *p* = 0.002, Site 1 vs. Site 3: adj. *p* = 0.006, Site 2 vs. Site 3: adj. *p* = 1.0). Community richness was not influenced by any significant interactions among site, veneer type and collection day.

### 3.2 Indicator Taxa and Community Composition

**FIGURE 3:**
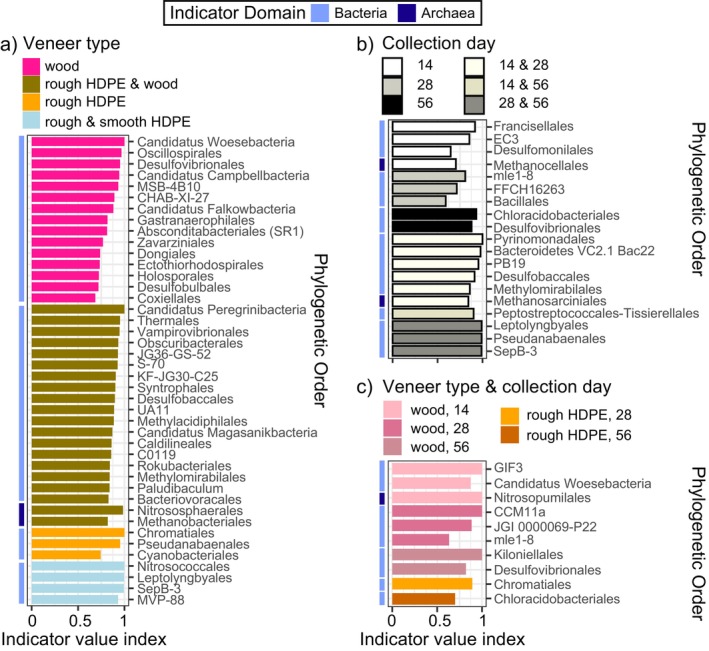
Microbial indicator taxa for (a) veneer type, (b) collection day, and (c) veneer type and collection day combined identified using indicator species analysis at the order phylogenetic level (*p* < 0.05, *N* = 3).

All veneer types and collection days had indicator orders except for smooth HDPE (Figure 3). Indicator orders occurred primarily for wood veneers either individually or in combination with rough HDPE (Figure 3a). Rough HDPE had only three indicator orders while smooth HDPE had no indicator orders by itself, but when combined with rough HDPE, plastic veneers had four indicator orders. Most indicators for the different veneer types were bacterial but the rough HDPE and wood veneer combined group had two archaeal indicator orders. Indicator orders occurred for individual and combined groups of collection days (Figure 3b). Early‐stage indicators also included orders from Archaea. The strongest indicator index values occurred for mid‐to‐late‐stage indicators where days 28 and 56 were grouped together. Indicator orders for veneer types and collection days combined also lacked any smooth HDPE indicators and were dominated by orders for wood veneers at all collection days (Figure 3c). Early‐stage (Day 14) wood veneer indicators included one archaeal order.

Dominant classes of microbial communities were similar regardless of veneer type or collection day (Figure S4). *Alphaproteobacteria*, *Gammaproteobacteria*, and *Bacteroidia* were the dominant classes in microbial communities on veneers regardless of the veneer type or collection day. *Alphaproteobacteria* ranged from 12% to 56% of the total community with a mean 30% relative abundance. *Gammaproteobacteria* ranged from 14% to 49% with 27% mean relative abundance. *Bacteroidia* ranged from 3% to 23% with 11% mean relative abundance. All other Classes were below 10% mean relative abundance.

### 3.3 Beta Diversity

**FIGURE 4:**
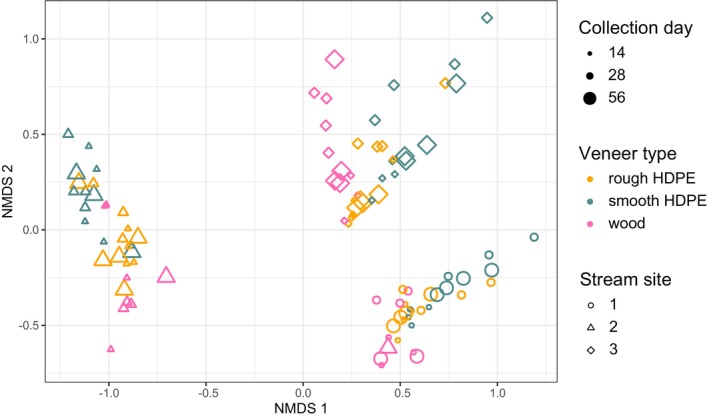
Non‐metric multidimensional scaling plot of microbial community composition in biofilms grown on three veneer types (rough high‐density polyethylene [HDPE], smooth HDPE and wood) incubated in headwater streams for up to 56 days (stress = 0.15, *N* = 3).

The community composition of biofilms was explained primarily by stream site and to a lesser extent by the veneer type and collection day (Figure 4). Our PerMANOVA tests indicated veneer type explained 8% of community composition variability; however, we did not document significant interactions between veneer type and time (Table 1). Subsequent pairwise comparisons indicated that beta diversity differed among all veneer types, but the difference was greatest between wood and both plastic veneers. Collection day explained the least amount of variability in composition (4.2%) and was the only factor to show significant differences in variance (homogeneity of multivariate dispersions, *p* = 0.015) (Table S3). Median group distances increased with collection day, suggesting community composition became more different over time (Table S3). We documented site‐specific differences in community composition as the main explanatory variable of community composition. Site explained 13% of the community composition variability, suggesting the potential for site‐specific environmental factors to govern microbial community composition. Subsequent, vector analyses demonstrated that stream water physicochemical parameters, including conductivity, pH, dissolved oxygen and temperature, also drove microbial community composition patterns (Table 2, Table S4).

**TABLE 1:** Permutational multivariate analysis of variance and pairwise comparisons for microbial community composition from different veneer types.

Overall beta diversity	*df*	Sum of squares	*R* ^2^	*F*	*p*
Veneer type	2	2.21	0.0843	5.44	***0.0001***
Collection day	2	1.10	0.0420	2.71	***0.0014***
Stream site	1	3.50	0.133	17.2	***0.0001***
Veneer type and collection day	4	0.975	0.0371	1.19	0.1737
Veneer type and stream site	2	0.719	0.0274	1.76	***0.0277***
Collection day and stream site	2	0.651	0.0248	1.60	0.0520
Veneer type and collection day and stream site	4	0.598	0.0228	0.735	0.9152
Residual	81	16.4	0.627		
Total	98	26.2	1.00		

Pairwise comparison of veneer types	Sum of squares	*F*	*R* ^2^	*p*	*p*.adj
Smooth vs. wood	2.70	4.11	0.0631	0.001	***0.003***
Smooth vs. rough	1.37	2.12	0.0298	0.011	***0.033***
Wood vs. rough	1.93	3.05	0.0469	0.001	***0.003***

*Note: p* values < 0.05 are bolded and italicised.

**TABLE 2:** Stream water environmental vectors correlated with beta diversity in non‐metric multidimensional scaling plot in Figure 4.

Environmental variable	NMDS axis 1	NMDS axis 2	*R* ^2^	*p*
Water temperature	0.48196	0.87619	0.3961	***0.001***
Dissolved oxygen	0.63554	–0.77207	0.5430	***0.001***
Conductivity	0.93062	–0.36599	0.7040	***0.001***
pH	0.97218	–0.23425	0.6504	***0.001***
Total organic carbon	0.52770	0.84943	0.0401	0.132
Chloride	−0.70165	0.71252	0.0948	***0.009***
Nitrate	−0.00711	0.99997	0.0877	***0.011***
Sulfate	−0.87838	–0.47797	0.2897	***0.001***
Phosphate	−0.69510	–0.71892	0.3256	***0.001***

*Note: p* values < 0.05 are bolded and italicised.

### 3.4 Biomass and Chlorophyll‐*a*

**FIGURE 5:**
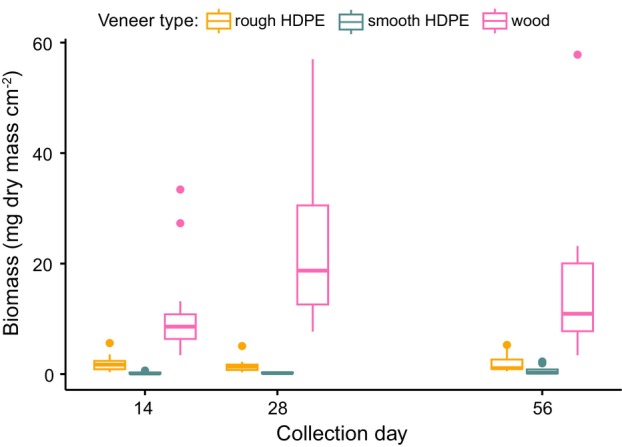
Biomass as dry mass of biofilm collected from three different veneer types (rough high‐density polyethylene [HDPE], smooth HDPE and wood) incubated in headwater streams for up to 56 days (linear model with post hoc Tukey contrast‐pairwise comparison tests, *α* = 0.05, *N* = 3).

Biofilm biomass differed among veneer types (F_4,95_ = 22.67, *R* ^2^ = 0.46, *p* < 0.001) (Figure 5). Wood had a greater biofilm biomass than either rough or smooth plastic (rough vs. wood: *β* = 14.63, *t* = 7.9, adj. *p* < 0.001, wood vs. smooth: *β* = 15.93, *t* = 8.2, adj. *p* < 0.001). There was no significant difference in biomass between the two plastic types nor between any of the collection days (all adj. *p* > 0.213). Wood veneers had approximately nine times more biomass than rough plastic veneers and about 40 times more biomass than smooth plastic veneers (wood: mean = 16.30 mg cm^−2^, S.E. = 2.23, rough: mean = 1.84 mg cm^−2^, S.E. = 0.25, smooth: mean = 0.40 mg cm^−2^, S.E. = 0.11).

**FIGURE 6:**
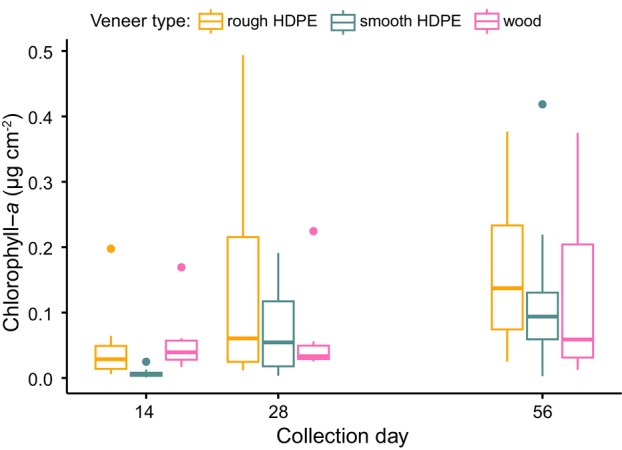
Chlorophyll‐*a* content in dry mass collected from three different veneer types (rough high‐density polyethylene [HDPE], smooth HDPE and wood), incubated in headwater streams for up to 56 days (linear model with post hoc Tukey contrast‐pairwise comparison tests, *α* = 0.05, *N* = 3).

Chlorophyll‐*a* concentrations differed by veneer type and collection day (F_4,95_ = 11.92, *R* ^2^ = 0.3061, *p* < 0.001) (Figure 6). Pairwise comparisons showed that on average chlorophyll‐*a* levels on smooth plastic veneers were lower than on either rough plastic or wood veneers (smooth vs. rough: *β* = −0.98, *t* = −3.5, adj. *p* = 0.004, wood vs. smooth: *β* = 0.82, *t* = 3.0, adj. *p* = 0.021). However, these patterns were not consistent across sampling dates and over time, median chlorophyll‐*a* levels on smooth plastic outpaced those on wood (Figure 5). Chlorophyll‐*a* levels on rough plastic and wood did not differ (adj. *p* = 0.97). Rough plastic had about 1.4 times as much chlorophyll‐*a* as wood and 1.7 times as much chlorophyll‐*a* as smooth plastic (wood: mean = 0.077 μg cm^−2^, S.E. = 0.016, rough: mean = 0.109 μg cm^−2^, S.E. = 0.021, smooth: mean = 0.063 μg cm^−2^, S.E. = 0.016). The chlorophyll‐*a* content of biofilms increased between earlier and later days in the experiment. We documented significant differences between the 14th and 28th collection day and between the 14th and the 56th collection day (day 28 vs. 14: *β* = 0.94, *t* = 3.5, adj. *p* = 0.005, day 56 vs. 14: *β* = 1.55, *t* = 5.7, adj. *p* < 0.001). However, there was no difference in chlorophyll‐*a* levels between the 28th and 56th collection day (adj. *p* = 0.15).

## 4 Discussion

Our work indicates that macroplastic pollution may alter the essential habitats created by biofilms and has the potential to influence the key functions performed by biofilm microbial communities in streams. Relative to biofilms colonising wood in this study, we documented significant differences between prokaryotic community diversity, associated taxa, and the total mass of biofilm colonising plastic substrates. Notably, the differences we documented between plastic and the natural substrate were sometimes associated with both plastic surface textures, but for others, the texture of plastic veneer surfaces had a greater influence on the responses we measured. Our findings also suggest the response of biofilms to novel substrates is dynamic, as we documented temporal variation on the influence of plastic pollution in some, but not all of our response variables. In sum, our results demonstrate that the physical structure and microbial diversity of biofilms among our substrates changed over time and were influenced by the texture of plastic veneers in stream ecosystems.

Macroplastic reduced the total number of microbial species found in biofilms and was associated with unique indicator taxa. We found that wood veneers had at least 17% more microbial species than either of the plastic treatments. Our results are similar to those documented by Shen et al. (2021), who found the same pattern by conducting a 40‐day in situ biofilm culture experiment in Xuanwu Lake, in Nanjing, East China. Their work examined microbial communities on cobble stone, wood, polyethylene terephthalate (PET) and polymethyl methacrylate (PMMA), and they found that wood had almost twice the number of species when compared to communities on plastic treatments.

Both the number of species and the identity of taxa associated with plastics can differ from natural substrates and may be consequential for nutrient cycling. In our study, plastic had four taxonomic orders specific to it: Nitrosococcales (an order of ammonia oxidizers), and Leptolyngbyales and *SepB‐3* which are both orders of Class Cyanobacteriia and contain photosynthetic, nitrogen fixing groups, and *MVP‐88* in Class Elusimicrobiota which are associated with spring and groundwater, soil and sediments and are under further study to determine nitrogen‐fixing potential (Méheust et al. 2020; Pedron et al. 2022; Chuvochina et al. 2023; Strunecký, Ivanova, and Mareš 2023). This suggests that polyethylene may harbour a unique community of nitrogen cycling taxa and supports prior research finding that nitrogen metabolism can play a critical role in polyethylene degradation (Peixoto et al. 2022).

Our results also suggest that the texture of plastic veneers influenced biofilm community species diversity and identity, as our rough HDPE had approximately 243 more unique species than smooth HDPE. This pattern may have been primarily attributed to increased surface area on the rough plastic, as greater numbers of species are expected in larger habitats per previously demonstrated species‐area relationships (e.g., Cairns and Ruthven 1970). Rough surfaces can ease microorganisms' initial attachment to surfaces by providing an adherence point for them (Rummel et al. 2017) and may also create heterogeneous flow environments that permit a greater variety of species to colonise the surface (Besemer et al. 2009). Lazcano, Kelly, and Hoellein (2024) documented a similar pattern of species diversity when comparing microbial communities on three plastic types, each having a different texture. They deployed low‐density polyethylene (rigid), low‐density polyethylene (film), foamed polystyrene, and wood substrates in the Chicago River, IL, USA. They documented that bacterial richness was two to three times greater on wood and foamed polystyrene in comparison to film and rigid polyethylene, although this varied by substrate surface size and successional stage. In addition to changes in richness, unique taxonomic orders were associated with the veneer textures in our study. While smooth HDPE had no indicator taxa orders, rough HDPE and wood veneers had indicator taxa orders spanning from the obligate anaerobe Desulfovibrionales, which contains many sulphate‐reducing species, to Nitrososphaerales, an ammonia‐oxidising class of Archaea (Kuever, Rainey, and Widdel 2005; Stieglmeier et al. 2014). Collectively, this indicates that smooth plastic was a poorer habitat that only supported a portion of the taxa found in prokaryotic biofilm communities growing on more textured surfaces.

Successional stage in our study was associated with unique indicator orders, which is expected for biofilm succession (Rickard et al. 2003), but our study was remarkable in that only wood veneers had unique orders associated with each successional stage. Smooth HDPE had no order indicators for successional stage and rough HDPE only had mid‐and‐late‐stage indicators. This implies that both the chemical composition of wood veneers and the microhabitats present in wood and rough HDPE surfaces can support assemblages that contain low phylogenetic resolution groups specific to a successional stage.

Microbial community evenness was also influenced by veneer texture. As expected, we found that microbial community evenness was greatest on veneers with rough surfaces, supporting our prediction that smooth surfaces would reduce community evenness. This pattern could be potentially due to competition, which is known to reduce species. In contrast to our expectations, we documented greater evenness on the rough plastic than the wood veneers. We expected wood to have greater evenness because we predicted that plastic chemical properties would inhibit the growth of certain taxa, allowing others to become more dominant. Similar findings were documented by Wang et al. (2021) who demonstrated that bacterial evenness was greater on polyethylene (PE) microplastics in comparison to PE and PP (polypropylene) veneers. These differences in bacterial evenness are potentially driven by the shape and texture of plastic. We also documented temporal changes in evenness during our experiment. Evenness declined in all veneer types throughout the experiment, and this decline was the greatest on smooth veneers. This is in contrast to findings from Cheng et al. (2020) who found that on meso‐debris (18 mm) in seawater, Pielou's evenness was positively correlated with time on HDPE, Poly L lactic acid (PLA), and glass over 66 days. Their work was conducted in laboratory conditions in stable flow environments with low‐nutrient seawater (< 0.04 μM nitrate and phosphate), whereas the dynamic flow environment and greater macronutrient availability in our field experiments may have boosted initial evenness.

Beta diversity in our study was strongly influenced by site‐specific characteristics. Site also influenced community richness but did not alter the relationship between veneer type and community richness. These results mirrored those documented by Vincent et al. (2022) who quantified beta diversity and richness of biofilms in six urban streams. They found that site‐specific factors had greater effects on microbial community composition than surface type and that OTU richness differed significantly among sites. Their study spanned urban systems across the U.S. and were subject to different climates and development patterns; therefore, these results were not particularly surprising. In our work, two other factors also influenced beta diversity in biofilms—time and veneer texture. Temporal shifts in beta diversity were also reported by Lazcano, Kelly, and Hoellein (2024) who found shifts in beta diversity over a six‐week sample period in an urban stream. They also demonstrated beta diversity differences between communities found on wood versus communities found on plastics, which was similar to our findings. However, in contrast to their work, we found differences in beta diversity between plastics with different textures. Together, our work suggests that site‐specific differences in biofilm community composition can be influenced by plastic pollution and that these relationships may change with time.

Regardless of veneer texture, natural substrates accrued more biofilm than did plastics in our experiment, but patterns were less clear for chlorophyll‐*a* concentration in the biofilms. We found at least nine times more biofilm dry mass on wood veneers than either of the plastics. However, we did not detect a significant difference in mass due to collection day, suggesting that this pattern was established within the first 2 weeks and was maintained through the experiment. Though it was outside the scope of this study, the difference in macroplastic‐based biofilms could have large effects on animal communities in streams. Biofilms provide essential habitat to stream organisms, often serving as a refuge from predators (Allan, Castillo, and Capps 2021). Our data indicate that if plastic replaces more natural hard substrates in streams, it may concomitantly provide different biofilm‐derived functional attributes in affected systems. Unexpectedly, we did not document statistically significant differences in biofilm mass between plastic treatments, indicating that increased surface area from the rough texture, did not equate to additional biofilm mass on veneers. This finding contrasts with work by Vincent et al. (2022) who documented greater biofilm mass on polystyrene, a plastic with more surface complexity, relative to PVC and tile that are characterised by smooth surfaces. Throughout our study, there were rain events that may have led to increased discharge that scoured our plastic treatments more severely than the wood treatments. However, we cannot assess this possibility, as we did not continuously measure water flow at each veneer type throughout our study. We did document differences in chlorophyll‐*a* concentration among substrates through time. Our findings contrast with those of Vincent et al. (2022) who did not detect an effect of surface type, but are supported by the results from Lazcano, Kelly, and Hoellein (2024) who documented chlorophyll‐*a* concentration shifts through time. However, our results did not present a clear pattern about the temporal effect of plastic on chlorophyll‐*a* concentration, especially when considered in context with findings from other studies. Thus, the influence of plastic pollution on photosynthetic organisms in biofilms warrants further study.

Our findings should be considered with several factors in mind. First, our work was conducted over an abbreviated period in relatively undisturbed streams. Over a much longer period of 167 days, Pedersen (1990) found that the abundance of bacteria was similar in biofilm that had developed in water pipes made from different materials including hydrophilic stainless steel, hydro‐phobic polyvinyl chloride (PVC), and PE. Although the length of our study was designed to cover the different stages of stream biofilm succession identified for other aquatic environments (Jackson, Churchill, and Roden 2001; Besemer et al. 2007) while avoiding major seasonal changes (Wang et al. 2022), our study was much shorter than Pedersen's (1990) (i.e., 56 days vs. 167 days). Thus, our findings might have changed in a longer study. Secondly, our work was conducted in rural streams with very limited macroplastic pollution. In contrast, many of the in situ studies examining the influence of plastic pollution on microbial communities in streams have been conducted in urban systems, where macroplastic pollution has been common for decades. In other words, plastics in our study sites represent novel habitats to colonise, and we anticipate that compared with streams having prior plastic pollution, these streams may foster fewer taxa already well‐suited to colonise plastics.

Global plastic production will continue and is projected to increase. Unless drastic changes are made, much of this plastic will enter rivers and streams and serve as a novel surface for biofilm development. Our work demonstrates that microbial richness and evenness in biofilm communities and the amount of habitat provided by biofilm mass are affected by plastic in comparison to naturally occurring substrates. Because biofilms are essential components of food webs and ecosystem processes, the effect of these compositional changes can influence their contribution to ecosystem processes. To predict the influence of plastic pollution on the structure and function of riverine systems, it is essential to estimate how diverse forms of plastic pollution will alter biofilm communities.

## Author Contributions

**Fabiola Lopez Avila:** conceptualization, investigation, writing –original draft, methodology, validation, visualization, writing – review and editing, data curation, formal analysis, project administration. **Krista A. Capps:** conceptualization, investigation, methodology, writing –review and editing, project administration, supervision, resources. **Raven L. Bier:** conceptualization, investigation, methodology, writing –review and editing, validation, visualization, funding acquisition, formal analysis, project administration, supervision, resources.

## Ethics Statement

The authors have nothing to report.

## Consent

The authors have nothing to report.

## Conflicts of Interest

The authors declare no conflicts of interest.

## Supporting information


Data S1.

Table S1.

## Data Availability

The data that support the findings of this study are openly available. Environmental data are included in the manuscript. The 16S rRNA genes sequences are openly available in the National Center for Biotechnology Information Sequence Read Archive at https://www.ncbi.nlm.nih.gov/sra, reference BioProject accession number PRJNA1053619.
