# Supplementary material for: Surface Texture of Macroplastic Pollution in Streams Alters the Physical Structure and Diversity of Biofilm Communities
Source: Environ Microbiol Rep. 2025 Apr 10;17(2):e70068. doi: 10.1111/1758-2229.70068 (PMC11982702; doi:10.1111/1758-2229.70068)
Supplement: Supplementary file 1 — Data S1. [file EMI4-17-e70068-s001.docx]

*Supporting Information for:* Surface texture of macroplastic pollution in streams alters the physical structure and diversity of biofilm communities

Fabiola Lopez Avila^1,2,3^, Krista A. Capps^1,2,4^, Raven L. Bier^1,5*^

^1^Odum School of Ecology, University of Georgia, Athens, GA 30602 USA

^2^Savannah River Ecology Laboratory, University of Georgia, Aiken, SC 29802 USA

^3^ flopezavila8@gmail.com, ORCID: 0009-0004-2137-3698

^4^ kcapps@uga.edu, ORCID: 0000-0002-9911-8644

^5^ rbier@srel.uga.edu, ORCID:0000-0003-1740-7184

*Corresponding Author: Raven Bier, Savannah River Ecology Laboratory, University of Georgia, PO Drawer E, Aiken SC 29802 USA. Phone: +1 803-725-9726, Email: [rbier@srel.uga.edu](mailto:rbier@srel.uga.edu)

**This file includes:**

Supplementary Tables S2 – S4

Supplementary Figures S1 – S4

Supplementary Table S1 is supplied as separate .xlxs file

Figure S1. Veneer placement in three blocks replicated at each stream site. Blocks within each site had a varied veneer order that was consistent across sites.

Table S1. Statistics from DADA2 sequence processing and curating of paired-end fastq files from amplicon sequence variants (ASVs). (Provided as a separate .xlxs file)

Figure S2. Rarefaction curves of samples indicating the relationship between sequence sample size and the number of unique amplicon sequencing variants (ASVs) or “species.”

Table S2. Mixed effects regression models of estimated richness and Pielou’s evenness of bacteria and archaea in biofilms grown on rough or smooth HDPE or wood veneers incubated in headwater streams and collected after 14, 28, or 56 days of incubation. Richness fitted model weighted *R*^2^ = 0.32. Pielou’s evenness fitted model weighted *R*^2^ = 0.53.

| Response | Fixed effects | | β | Std. Error | p |
| --- | --- | --- | --- | --- | --- |
| Bacterial/archaeal richness | (Intercept is wood veneer) | | 1145 | 49 | **< 0.001** |
|  | Veneer surface (rough) | | -138.8 | 82 | 0.090 |
|  | Veneer surface (smooth) | | -381.9 | 83 | **< 0.001** |
|  | Collection day | | -3.79 | 1.3 | **0.005** |
|  | Veneer surface (rough) × Collection day | | -2.91 | 2.3 | 0.198 |
|  | Veneer surface (smooth) × Collection day | | -2.05 | 2.3 | 0.370 |
|  |  | |  |  |  |
|  | (Intercept is rough HDPE veneer) | | 1007 | 49 | **< 0.001** |
|  | Veneer surface (smooth) | | -243.0 | 83 | **0.003** |
|  | Veneer surface (wood) | | 136.4 | 92 | 0.139 |
|  | Collection day | | -6.72 | 1.3 | **< 0.001** |
|  | Veneer surface (rough) × Collection day | | 0.863 | 2.3 | 0.706 |
|  | Veneer surface (smooth) × Collection day | | 2.97 | 2.5 | 0.226 |
|  | *smooth HDPE veneers* | |  |  |  |
|  | (Intercept) | | 731.4 | 60 | **< 0.001** |
|  | Collection day | | -5.37 | 1.7 | **0.001** |
|  | *rough HDPE veneers* | |  |  |  |
|  | (Intercept) | | 984 | 62 | **< 0.001** |
|  | Collection day | | -6.30 | 1.7 | **< 0.001** |
|  | *wood veneers* | |  |  |  |
|  | (Intercept) | | 1130 | 69 | **< 0.001** |
|  | Collection day | | -4.52 | 1.8 | **0.012** |
| Bacterial/archaeal Pielou’s evenness | (Intercept is wood veneer) | 90.44 | | 0.26 | **< 0.001** |
|  | Veneer surface (rough) | 0.848 | | 0.42 | **0.045** |
|  | Veneer surface (smooth) | -2.58 | | 0.43 | **< 0.001** |
|  | Collection day | -0.004 | | 0.01 | 0.591 |
|  | Veneer surface (rough) × Collection day | -0.006 | | 0.01 | 0.578 |
|  | Veneer surface (smooth) × Collection day | -0.126 | | 0.01 | **< 0.001** |
|  |  |  | |  |  |
|  | (Intercept is rough HDPE veneer) | 91.53 | | 0.26 | **< 0.001** |
|  | Veneer surface (smooth) | -3.639 | | 0.44 | **< 0.001** |
|  | Veneer surface (wood) | -1.309 | | 0.50 | **0.009** |
|  | Collection day | -0.016 | | 0.01 | **0.020** |
|  | Veneer surface (rough) × Collection day | -0.113 | | 0.01 | **< 0.001** |
|  | Veneer surface (smooth) × Collection day | 0.016 | | 0.01 | 0.212 |
|  | *smooth HDPE veneers* |  | |  |  |
|  | (Intercept) |  | |  |  |
|  | Collection day |  | |  |  |
|  | *rough HDPE veneers* |  | |  |  |
|  | (Intercept) |  | |  |  |
|  | Collection day |  | |  |  |
|  | *wood veneers* |  | |  |  |
|  | (Intercept) |  | |  |  |
|  | Collection day |  | |  |  |


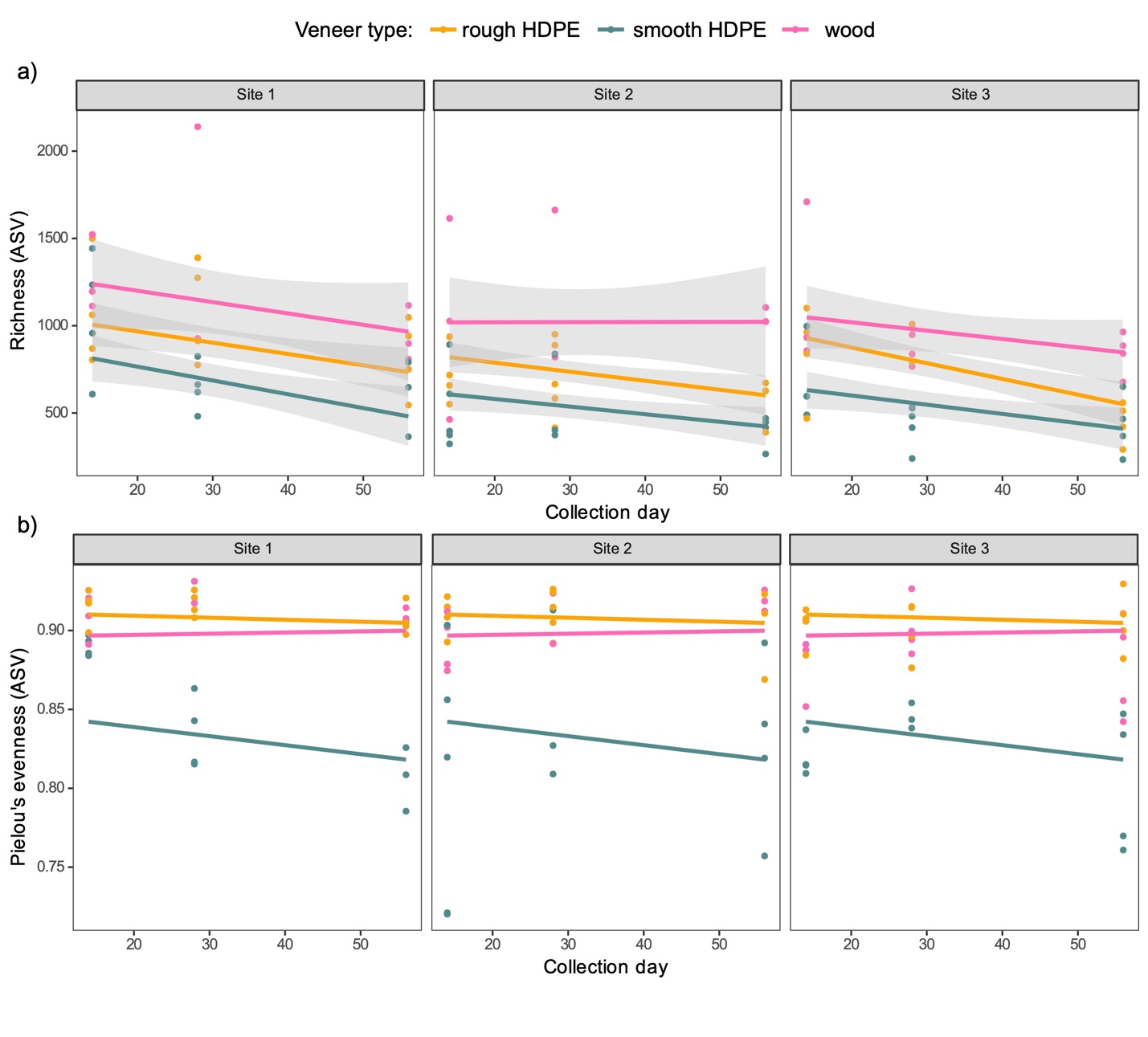


Figure S3. Mixed effects regression models of biofilm microbial community a) richness and b) Pielou’s evenness on rough or smooth HDPE or wood veneers incubated in headwater streams and collected after 14, 28, or 56 days of incubation. Observed values are points and regression lines are predicted values. Shaded grey zones depict 95 % confidence intervals which are not visible for evenness. Each panel shows a different stream site.

Figure S4. Bacterial and archaeal classes of the 500 most common amplicon sequence variants from biofilms grown on rough or smooth HDPE or wood veneers incubated in headwater streams and collected after 14, 28, or 56 days of incubation.

Table S3. Multivariate homogeneity of groups dispersions based on the Bray-Curtis distance dissimilarities for microbial community composition at different collection days with distance to centroids within factors and between groups

|  | Df Sum of Sq Mean Sq F N. Perm p-value | |
| --- | --- | --- |
| Veneer type  Residuals  Collection day  Residuals  Stream number  Residuals | 2 0.0138 0.00690 2.03 999 0.136  96 0.325 0.00338  2 0.0280 0.0140 4.12 999 ***0.015***  103 0.350 0.00339  2 0.0499 0.0249 3.14 999 0.054  103 0.817 0.00793 | |
|  | | |
| **Within groups**  Average distance  Collection day to median | | **Between groups**  Average distance  Collection day to median |
| 14 0.505  28 0.528  56 0.545 | | 14 vs. 28 0.148  14 vs. 56 0.211  28 vs. 56 0.161 |

Df = degrees of freedom. N. Perm = number of permutations

*p-values <0.05 are bolded and italicized

Table S4. Environmental variables collected from three stream sites on each sampling day

|  | **Site 1** | | | **Site 2** | | | **Site 3** | | |
| --- | --- | --- | --- | --- | --- | --- | --- | --- | --- |
| Collection day | *14* | *28* | *56* | *14* | *28* | *56* | *14* | *28* | *56* |
| Stream width (m) | 1.434 | 1.475 | 1.592 | 1.031 | 1.420 | 1.035 | 1.360 | 1.843 | 1.725 |
| Average stream depth (cm) | 3.05 | 6.13 | 6.43 | 8.51 | 7.20 | 9.98 | 5.71 | 7.21 | 6.54 |
| Average veneer depth below water surface (cm) | 1.09 | 2.95 | 3.73 | 3.05 | 3.50 | 5.35 | 3.12 | 3.33 | 4.75 |
| Water temperature (∘C) | 21.4 | 20.2 | 21.4 | 20.6 | 19.8 | 20.9 | 23.8 | 21.7 | 23 |
| DO (%) | 95.2 | 94.9 | 92.4 | 90.7 | 91 | 89.1 | 91.6 | 92.1 | 89 |
| Conductivity (μS cm^-1^) | 23.3 | 22.5 | 21.8 | 11.8 | 11.7 | 13 | 14.4 | 15.2 | 18.2 |
| pH | 6.94 | 6.36 | 5.98 | 5.51 | 5.06 | 4.96 | 6.18 | 6.09 | 5.78 |
| TOC (mg L^-1^) | 2.85 | 3.16 | 6.62 | 3.50 | 2.25 | 4.68 | 2.38 | 2.38 | 5.48 |
| Chloride (mg L^-1^) | 1.49 | 2.12 | 2.59 | 2.64 | 1.38 | 3.33 | 1.13 | 2.27 | 2.82 |
| Nitrate (mg L^-1^) | 0.04 | 0.11 | 0.03 | 0.29 | 0.04 | 0.04 | 0.40 | 0.33 | 0.11 |
| Sulfate (mg L^-1^) | 0.16 | 0.20 | 0.21 | 0.36 | 0.12 | 0.29 | 0.20 | 0.13 | 0.13 |
| Phosphate (mg L^-1^) | 0.03 | 0.09 | 0.04 | 0.08 | 0.09 | 0.11 | 0.11 | <0.01 | <0.01 |
